# Supplementary material for: Are carbon emissions trading and green financial instruments synergistic? -Comprehensive quantitative research based on content analysis
Source: PLoS One. 2024 Mar 7;19(3):e0298601. doi: 10.1371/journal.pone.0298601 (PMC10919601; doi:10.1371/journal.pone.0298601)
Supplement: S1 Data — (ZIP) [file pone.0298601.s001.zip › Data/地级市Z_F工作报告.zip!/║┌┴·╜¡/║╫╕┌╩╨/└┤╫╘╣1⁄2╓┌║┼í╛┬φ┐╦╩2╛▌═°í┐_2007.docx]

各位代表：

　　现在，我代表鹤岗市人民政府向大会作政府工作报告，请予审议，并请各位委员提出意见和建议。

　　一、四年政府工作回顾

　　第十三届政府任期四年来，在省委、省政府和市委的正确领导下，坚持以科学发展观为统领，牢牢把握经济社会发展大局，不断完善工作思路，硬化发展措施，强化工作落实，开创了经济社会发展新局面。

　　——经济全面快速发展，结构调整扎实推进。四年中，经济总量由2002年的70.1亿元跃升到131亿元，增长86%；财政收入由6.06亿元跃升到16.1亿元，增长2.7倍；累计完成固定资产投资125亿元，实施重点项目120项；规模以上工业增加值由15亿元增加到34.6亿元，增长1.3倍；农业增加值由6.1亿元提高到11.5亿元，增长88.5%，粮食产量达到12.38亿斤，增长82%；社会消费品零售总额由 25.1亿元发展到36.76亿元，增长46.45%；金融机构各项存款余额由101.8亿元增加到161.7亿元，增长58%。食品工业基地粗具规模，煤电化能源工业基地加速建设。

　　——社会事业全面进步，各项工作取得新成绩。基础教育成绩优异，职业教育、高等教育健康发展，办学条件明显改善，消灭了中小学D级危房，新建了三中教学楼、职教中心等8万余平方米教学设施。卫生事业快速发展，新建了疾病预防控制中心、传染病院，疾病预防控制和处置突发公共卫生事件的能力进一步增强，农村合作医疗试点工作进展顺利。广播电视基础设施建设水平明显提高，投入近2000万元实施了有线电视网络数字化改造工程。文化事业长足发展，群众文化活动丰富繁荣。人口稳定低生育水平，妇女儿童工作得到加强。金融、监察、环保、民族、宗教、外事、侨务、审计、民政、双拥、人口、国防、人防、档案、司法、人事、物价、统计、地震、残联、老龄等都取得了显著成绩。

　　——基础设施全面加强，城乡面貌焕然一新。水利建设成效显著，大江大河、中小河流防洪除涝建设及病险水库除险加固等重点水利工程全面完成，防洪除涝标准明显提高。绥名公路、哈肇公路、鹤北支线、鹤大公路零公里至南风井拓宽工程和617公里通村、通乡公路相继建成。投资3.71亿元，进行了城乡电网改造。建设城市住宅231万平方米，新改建城市主次干道50条，硬化街巷道路129条，新建光宇桥、兴安桥、庆丰桥等城市桥梁。实施了沉陷区治理一、二期工程，新建南翼、光宇、德政三个小区9744户住宅。完成供热二期工程，新增供热能力100万平方米。近三年，投入3000多万元进行城区防洪工程建设，防洪标准提高到20年一遇。新建改造了振兴广场、人防三期、体育场、影剧院等项目，城市功能更加完善。

　　——各项改革全面深化，对外开放不断扩大。农村各项改革扎实推进。国有企业办社会职能逐步分离，共接收企业办医院5所、中小学23所。企业改革不断深化，并轨任务基本完成。粮食流通体制改革取得重要进展，粮企改革完成阶段性任务。教育改革稳步实施，完成了中小学人事制度改革。财政实施了政府集中采购、国库集中支付制度改革。工行、建行等国有商业银行完成了股份制改革，农村信用社改革初步完成，城联社改革正在实施。对外经贸合作向纵深发展，外贸进出口持续快速发展，年均增长30%；对俄经济技术合作项目27项。招商引资成果丰硕，到位资金152亿元，引进项目1385个，其中超亿元项目11个。

　　——人民生产生活条件全面改善，生活水平显著提高。城镇居民人均可支配收入达到8400元，比2002年增长74.5%；在岗职工平均工资由2002年的8475元达到现在的1.6万元，增长近一倍；农民人均纯收入达到 3781元，翻了一番。城市居民人均住宅建筑面积22.1平方米，农村22.3平方米，居全省上游水平。累计实现就业再就业10万余人次，城镇登记失业率控制在4.3%以内。养老保险金、失业保险金按时足额发放；提高了城市低保标准，做到了应保尽保；城镇职工基本医疗保险达到32.6万人；为61397名各类企业并轨职工发放经济补偿金54237万元；困难群体得到更多的关心和救助。

　　——政府自身建设全面加强，职能转变不断深入。积极进行政府管理机制和制度创新。建立了十项专项制度；组建了人民办事中心，把担保中心、国有资产运营中心办成了独立法人实体，建立了项目建设服务中心，改善了发展环境，方便了人民群众。深化行政审批制度改革，推行阳光政务，坚持依法行政，提高了政府办事效率。

　　我们虽然取得了一定成绩，但还面临一些突出问题和困难。一是经济发展中的矛盾依然突出。经济总量小，发展速度慢；产业层次低，附加值低；产业结构不合理，煤炭独大，发展空间小，消化市场风险能力弱。二是社会矛盾仍然突出。社会困难面大，企业历史遗留问题多；基础设施、社会事业欠帐多，发展需求大；安全生产任务重，社会稳定压力大。三是发展经济、项目建设难度加大。国家宏观调控力度加大，项目建设门槛提高、条件从严、难度加大。四是政府自身建设差距较大。按照人民群众的要求和新形势、新任务的需要，政府工作人员的知识、能力、作风还有很大差距，提高政府自身建设水平的任务繁重。

　　各位代表，刚刚过去的2006年是我们成果丰硕、持续快速发展、各方面工作都取得优异成绩的一年。

　　一是主要经济指标大幅增长。在上年经济高速运行的基础上又实现了高速增长。地区生产总值预计实现131亿元，比上年增长13%，一、二、三产业分别增长11.5%、15%、11.5%；全口径财政收入实现16.1亿元，增长17.7%，其中地方一般预算收入实现7.03亿元，增长24.3%；固定资产投资预计完成43.3亿元，增长33.8%；招商引资到位资金61.99亿元，增长37%；外贸进出口额完成2706万美元，增长91.2%；完成规模以上工业增加值34.6亿元，增长16.9%，利税实现9亿元，增长30%。

　　二是工业项目建设成效显著。组织“项目建设年”攻坚活动。大项目建设取得重大突破。北台钢铁集团投资60亿元的百万吨化肥、中国化学工程集团投资170亿元的大型煤化工多联产项目一期120万吨甲醇前期工作进展顺利，都已进入国家环保局审批程序，今年有望开工建设；兴泽集团和江苏格兰特集团合作投资10亿元的100万吨玉米深加工项目，一期30万吨已建成投产，二期70万吨已开工建设；投资20亿元的电厂二期工程今年上半年将投产发电；投资10.2亿元的鸟山煤矿项目已完成部分地面设施建设。高新技术、非煤产业项目形成规模。木糖醇、圣元乳业扩产、米糠深加工、康维生物保健食品及为鹤矿集团配套加工等一批新项目、高科技项目正在实施。后续项目储备充足。200万千瓦电厂三期、石墨深度开发、180万千瓦龙江电站、林木及食品加工等一批大中型项目已在积极运作中。老企业改革改造项目有较大进展。完成了工业布厂二轮租赁、禾友农药对外招商、圣尔药业扩产改造等一批改革改造项目。

　　三是新农村建设开局良好。农业结构不断优化。粮食总产创历史新高；畜牧业稳步发展，实现增加值2.67亿元，增长8.9%；奶牛、肉牛饲养量分别增长16%、10%。产业化水平提升。龙头企业实力明显增强，鹤鸣米业、泰丰米业和人和米业三户企业生产的大米被确定为全省免检产品；万源油脂公司的米糠油精深加工产品在全省率先打入国外市场；鹤鸣米业老工业基地改造项目一期工程四级米糠油已投产。农村面貌有新改观。当年筹资2亿多元，建设25个新农村试点村屯道路、自来水等重点项目，解决了18个村人畜饮水安全问题，试点村一步跨越、面貌巨变。

　　四是城乡基础设施建设全面加强。城市第三轮总体规划已获国家批准；启动了棚户区改造、煤层气民用扩容、松花江大桥、鹤名铁路、城市污水处理等一批重点项目前期工作。突破拆迁难点，促进了建筑市场迅速回升。完成投资1856万元修建了光宇路等11条城市主次干道和光宇桥、兴安桥、鹤兴桥；供热二期工程完成投资3800万元，新增供热能力100万平方米；市财政直接投入1360万元，建设了62条高标准的街巷道路，维修了300多条街巷道路；投资1600万元整治“五沟、一河、一库”和城市易涝区，基本解决了居民被淹问题。投资1.37亿元建成通村通乡公路320公里，投资2.1亿元建成99.5公里绥滨至名山公路；投入5400万元建设防洪工程和修复水毁工程；投入3500万元完成120公顷土地复垦和矿山地质公园等环境治理项目。

　　五是社会事业建设步伐加快。科技工作创新发展，推进了石墨综合利用、煤矿生产安监系统等15项科技合作项目。卫生保健水平继续提高，计划免疫工作不断强化，各类疾病防治水平有新提高，社区卫生服务网络基本形成，人口覆盖率达73%。广播电视事业实现新进步，投资600多万元完成600公里光缆建设，开通了三套省台广播节目和31套数字电视节目，电视节目已达百套以上。竞技体育水平进一步提高，参加省第十一届运动会名列金牌总数和团体总分第五名，居四煤城之首。成功举办了中国“俄罗斯”年活动。筹资3640万元，改造完成了24000平方米D级学校危房，新建了6000平方米职教中心教学楼，市传染病院正式启动，完成了市影剧院、万人体育场改造工程，购置特种消防设备、改造消防二中队、建成使用特警武警新营区，广播电视演播大厅、市老年活动中心工程在建，年初人代会确定事关社会事业发展的10件大事件件得到落实。

六是安全生产、社会治安、信访工作稳定。以解决问题为治本措施，全面加强信访工作，筹资2715万元解决企业遗留问题，使一大批信访积案得到处理，到市集体访、个体访下降21%和16.8%，进京到省集体访下降50%和68.8%。坚持不懈地强化安全生产，安全形势稳定。积极推进“平安鹤岗”建设，保持严打高压态势，实施了重点专项整治，刑事案件发案率下降5.3%，破案率上升8.4%，营造了良好的治安环境。

　　七是人民群众得到了实惠。人民收入持续增加，城镇居民人均可支配收入同比增长12.2%，在岗职工平均工资增长19.6%；农民人均纯收入增长9%。新增就业2.53万人，实现再就业2.1万人次，城镇登记失业率控制在4.4%以内。市财政支付2000万元为党政机关事业单位人员普调了工资，增资1500万元。实行热费补贴改革，由原来的7000人增加到12000人。社会保障体系不断完善，养老保险、失业保险金按时足额发放，提高了1.96万名企业离退休人员的养老保险待遇；筹资400万元，开展了慢性病门诊治疗，提高了大病救助标准；财政每年支付保费1100万元，将200余户关停破产企业 11286名退休职工纳入基本医疗保险。投入再就业资金8227万元。全年发放低保资金超亿元，新增资金4000万元将低保月人均补差额由65元提高到80元，为城市低保户发放热费补助。筹集资金300余万元，救助了4470名贫困学生；启动专项救助资金，对500余户特殊困难群体进行救助；减免了21274名学生的学杂费，为18274名农村学生发放了补助金；对未参保的集体企业退休职工按低保标准发放生活费。投资1.5亿元，年初市政府对市民承诺的廉租房等20件实事全部完成。

　　八是政府自身建设取得新成效。进一步转变政府职能，减少审批项目115项，继续加强完善政府机制制度建设，在前年实施十项制度的基础上，去年新设立十项专项资金，出台了专项资金使用管理、廉租房建设管理、河道建设管理、街巷道路建设管理、非税收入管理等专项制度。人民办事中心运行良好，一年来，受理各类业务12万件，办结11.7万件，办结率98%，办事时限压缩60%以上，办事效率平均提高2.5倍，收费标准普遍降低，仅15个涉及项目建设和群众生活的收费项目平均降幅达51%，收费总额比上年增长34%，人民群众满意率为95.7%。加强社区建设，使社区成为党和政府联系人民群众的桥梁纽带，畅通了人民群众诉求渠道，强化了对群众的服务。政府注重在涉及人民切身利益、完善城市功能、政府职能转变的大事、实事上探索建立长效机制，使城市的组织体系、管理机制和社会功能趋于完善、规范和成熟。

　　过去的四年，特别是2006年我们取得了巨大成就，创造了新的辉煌，这是省委、省政府和市委正确领导的结果；是人大依法监督、积极支持的结果；是政协民主监督、参政议政的结果；是多届班子长期努力厚积薄发的结果；是鹤矿集团、宝局、鹤北局、电厂等域内各大经济单元、驻鹤各单位和社会各界共同努力、共同创造的结果；是全市人民万众一心、艰苦奋斗的结果；是国内外所有鹤岗朋友鼎立相助的结果。我代表市政府向市委、人大、政协几大班子，向市内各大经济单元和中省直驻鹤各单位领导，向历届班子的老领导，向全市广大工人、农民、知识分子和各级干部，向所有鹤岗的劳动者和建设者，向驻鹤解放军指战员、武警官兵和公安干警，向关心和支持我市建设的各方朋友，表示崇高的敬意和衷心的感谢！

　　二、今后五年的总体构想

　　今后五年是鹤岗经济社会发展十分重要的关键时期，必须抢抓机遇，又好又快发展，全面实现“十一五”规划目标、振兴老工业基地任务和市十次党代会绘制的宏伟蓝图。

　　今后五年的总体工作要求是：以邓小平理论和“三个代表”重要思想为指导，全面贯彻落实科学发展观，认真贯彻中央、省委、省政府和市委的各项重大部署，继续实施工业立市和项目牵动战略，强化招商引资，开展全民创业，优化发展环境，转变增长方式，闯出一条老工业基地创新发展、资源型城市可持续发展、欠发达地区跨越发展、边疆城市开放发展、革命老区和谐发展的新路子，着力打造煤电化能源基地、绿色优质食品基地，对俄经贸合作区、边疆生态旅游区，继续发展优势传统产业和高新技术产业。推动政治建设、文化建设、社会建设与经济建设协调发展。全面履行政府职能，为实现城市创新转型、人民富裕幸福的目标，经济社会又好又快发展，构建和谐社会做出卓有成效的努力。

　　（一）落实科学发展观，实现经济又好又快发展

　　要以经济发展为中心，坚持从实际出发，落实践行科学发展观，努力把经济发展纳入良性科学轨道，实现又好又快发展。一是坚持高效发展。努力转变经济增长方式，做到以经济效益为中心，不断提高经济发展质量。要大力推进科技进步和适用科技推广，努力增加经济发展的科技含量。要进行产品深度开发，提高附加值。要实施品牌战略，增强产品的市场竞争力。二是坚持快速发展。作为欠发达地区，我们的主要矛盾是经济总量小、发展速度慢。没有规模、没有速度，就谈不上发展，更谈不上科学发展、又好又快发展，必须全面实现市党代会确定的经济发展目标，利用一切有利机遇和条件，努力发展得更快一些。三是坚持清洁发展。作为后发地区，我们必须吸取一些地区的经验教训，高度重视环保和生态，不蹈先污染后治理、边污染边治理的覆辙，坚决做到对环境有污染的项目不上，新上项目必须在排放上达标；对原有企业、城市污水、垃圾加快治理。五年内，市内要建成污水处理厂、垃圾处理厂，小烟囱再减少30%，改善大气质量；争取流经城区的小鹤立河、石头河等主要河流实现清水过境，保证鹤岗在经济快速发展的情况下，天蓝水绿、空气清新、生态优良。四是坚持节约发展。加强宏观调控，减少高耗能、高耗资源的企业和项目。要加强技术改造，引进新工艺、新设备，努力使能耗和资源消耗降低20%。要加强企业内部管理，减少跑、冒、滴、漏和损失浪费。要大力发展循环经济，实现变废为宝。五年内，煤矸石、粉煤灰、煤层气、煤焦气、工业废水、木材枝桠、稻壳、秸秆综合利用率和再利用率提高一倍。五是坚持创新发展。搞好自主创新、技术研发和技术引进、适用技术推广，应用高新技术改造传统产业，装备新产业，走新型工业化道路，真正把经济发展转到依靠科技进步的轨道上来。五年内，科技贡献率要提高到45%。六是坚持协调发展。要坚持经济社会发展相协调，全面发展社会事业。按照科学发展观和构建和谐社会的要求，统筹经济与社会，物质文明、精神文明、政治文明协调发展。努力增加社会事业投入，人民群众生活质量明显提高，社会和谐提高到较高水平。七是坚持惠民发展。坚持以人为本、发展惠民的宗旨和原则，让改革发展的成果全体人民共享。努力培植财源和就业岗位，增加人民群众和机关干部的收入，提高人民群众的物质文化生活水平；加快社会保障体系建设，逐步提高保障水平；加快发展完善社会事业、城乡基础设施和社区服务体系，解决好人民群众最关心、最直接、最普遍、最具体的现实问题；加强对社会弱势群体、困难群体的及时、有效救助，缩小贫富差距，提高社会文明和谐水平。八是坚持安全发展。坚持“安全第一、预防为主、综合治理”的原则，高度重视、切实加强安全工作，全力保障人民生命财产安全。要加大安全生产投入，加快安全设施改造建设，加强教育和监督管理，强化全社会安全意识，提高事故救援水平和应急控制能力。加强社会治安综合治理，确保为全市人民提供安全、平安的生产生活环境。九是坚持可持续发展。要遵循自然规律、经济规律和社会发展规律，既实现现实发展，更要打基础、攒后劲。要更加注重对经济社会发展有长远拉动支撑作用的大项目建设，更加注重社会事业发展，更加注重基础设施建设，更加注重事关人民生活的配套项目建设，更加注重精神文明、城市文化等软环境建设，更加注重人才的培养和人才队伍建设。

　　（二）优化经济产业结构，加速城市创新转型

　　市委确定的城市创新转型的总体战略切中实际、远见卓识，既是我市长远发展的必然，又是现实加快发展的必须，要扎实落实、加快实施。作为以煤炭采掘为主的资源型城市，实现城市创新转型，要坚持从我市的比较优势出发，大力发展接续替代产业，从根本上优化经济结构，要抓好八项重点工作。

　　一是由现有煤炭资源的短期规模开发向发展煤炭后备资源和控制长期开发转型。煤炭是不可再生资源，现实技术条件下，回采率、利用率和产业产品附加值都很低。按已探明的可采储量，现有规模开发的年限有限，要以延长煤矿服务年限为重点，大力采用新技术、新工艺、新设备，提高回采率，控制开采规模，延长开采时间。要加快鸟山煤矿、新华煤田的开发建设；加大后备资源的探查力度，积极寻找新煤田。要积极争取对萝北大马河和绥滨永祥、福兴煤田进行详查，努力建设百年以上煤田。

　　二是由以煤炭采掘为主产业向以煤炭深加工为主产业转型。要加快煤炭加工转化速度，延长产业链条。要大力发展煤电、煤焦、煤化工、煤层气、粉煤灰和煤矸石利用等工业产业，使之成为支撑市矿经济的主体产业，实现煤炭采掘和深加工主辅换位。五年内，要使煤炭就地加工转化率达到60%以上，用加工增值效益替代部分原煤效益，冲减原煤产量。

三是由煤炭产业为主向非煤产业转型。要以非煤产业为主要替代产业重点开发。鹤岗既是煤城，有丰富的煤炭资源，又位居三江平原、小兴安岭，与俄远东地区接壤，有丰富的农牧、林木和多种矿产资源、优势旅游资源。要加快这些资源的开发，培育新的主导产业，使非煤产业逐步成为市域经济的主导产业、煤经济的替代产业。要重点发展米、油、肉、乳、玉米化工等优质食品工业和粮食化工业，发展林木加工业，发展石墨等金属非金属矿产加工业，发展医药、机械、建材等加工业，发展现代物流业和旅游业。五年内，非煤产业总量要翻一番，逐步实现煤与非煤产业的主辅换位。

　　四是由传统产业向高新技术产业转型。我市的制酒、机械、建材、医药等传统产业有一定基础和市场优势，要采取综合措施做强做大。要根据市场需求和生产要素优势，积极发展高新技术产业，大力开发新品种，做强医药业，大力发展精细化工、电子等新兴产业，开辟新的经济领域。

　　五是由单一主体产业向发展循环经济转型。随着经济发展、资源价值增值，过去很多废弃物已成为今天的宝贵资源。要大力发展废弃资源的开发利用，发展循环经济，补充主体产业。大力发展煤矸石发电、煤层气民用和工业加工、秸秆发电和秸秆饲料、利用农村粪便垃圾发展沼气、粉煤灰等工业废渣制砖制水泥、煤矿矿井水净化、生产生活废水综合利用、垃圾无害化处理等。五年内，循环经济总量要增长两倍。

　　六是由一次性资源开发向可再生资源开发利用转型。一次性资源终有枯竭之时，开发可再生资源，才能永续利用和可持续发展。要保护好耕地资源，长期保持农业发展优势。加大黑龙江、松花江水资源的开发利用，增加水灌面积，提高农畜产品产量，扩大农畜产品加工业规模。五年内，争取建设三个灌区，新增水田面积68.7万亩，新增粮食产量5亿斤。要抓紧前期工作，建设180万千瓦龙江水电站；着手研究风能开发利用，在萝北、绥滨建设风力发电站；要加强林业资源的保护开发，实现良性循环、永续利用。

　　七是由资源初加工向精深加工转型。我市已有的资源加工产业多是初加工，原油（粕）、原煤、原肉、原粮（米）、原木、原奶（干粉）产品居多，附加值低，资源消耗大，精深加工潜力巨大、效益巨大。要重点发展大豆系列精深加工、生猪肉制品以及毛、皮、骨、血生物制药等精深加工，发展玉米、水稻粮食食品精深加工、粮食化工精深加工，发展精细煤化工、石墨、医药等高端产品，做到新产业、精产品、高效益、大贡献。

　　八是由注重利用市内国内资源、市场向开发利用国内国际资源、市场转型。借助经济全球化的发展趋势和地处东北亚中心地带、接壤俄远东地区的地缘优势，大力开发利用俄罗斯的自然资源和俄、日、韩等国际市场，开发市外粮牧、林木等加工资源，借船出海，借鸡生蛋，补充自己，发展自己，实现双赢。

　　（三）加快城乡基础设施建设，全面发展社会事业

　　要加快城市建设。治理沉陷区，改造棚户区，辟建城市新区，建设工业园区，完善城市社区，进一步提高城市功能，改善城市条件。五年内，老城区改造要上水平，城市新区开发建设成规模。完成城市主次干道建设，硬化全部街巷道路，新建城市住宅250万平方米以上，新增城市绿地5万平方米，投资2.14亿元建设供热三期工程，新增供热面积300万平方米；投资5亿元，加快城乡电网建设；建成绥滨松花江大桥、鹤名公路复线、名山二港区，力争完成兴东至嘉荫河口82公里国防公路、鹤佳至鹤伊23公里城市西外环、哈肇至鹤伊21公里城市东北外环等建设任务，形成城市交通大外环。

　　要加快农业农村基础设施建设。以新农村建设为统领，加快农田水利、村镇道路、农村饮水等基础设施建设。五年完成通乡通村公路1200公里，实现村村通水泥路；完成150个村屯人畜饮水工程，解决7.5万人的饮水安全问题；力争完成3个水库建设，新增供水能力4.1亿吨。

　　要全面发展社会事业。优先发展教育，做到普通教育、职业技术教育、高等教育全面协调发展。加快卫生事业发展，推进新型农村合作医疗，实现全覆盖，完成乡镇卫生院改造；社区卫生医疗服务机构全部覆盖，规范化、标准化率达80%；医疗急救、传染病预防救治、公共卫生突发事件处置能力大幅增强。完善劳动保障和社会救助体系，弱势群体利益得到有效维护。提高广播电视专业化、现代化水平，有线电视实现数字化整体转换。加快科技创新和科技进步步伐，实施城市、企业、农村信息化工程，提高全社会信息化水平。加强安全生产和社会治安综合治理，提高信访工作水平。同时要加快各项事业发展。

　　（四）坚持利民惠民，构建和谐社会

　　坚持发展富民、发展惠民、发展利民、发展成果由全体人民共享的基本原则。一要努力发展经济，扩大就业。要继续实施创业就业优惠政策，增加就业容量，实现充分就业；要实施最低工资标准，并随着经济的发展不断提高；要搞好就业登记管理，保障就业人员特别是农民工合法权益；要增加购买公益性岗位，解决大龄下岗失业人员就业问题。二要让利于民。减少收费项目，降低收费标准，在拆迁、征地、企改等重点领域，优先保证人民群众和职工利益。三要搞好扶贫帮困和社会救助工作。继续深入开展城市“一帮一扶贫解困工程”，坚持社会捐助救助机制，用好政府专项救助资金，逐步提高低保、抚恤和专项救助水平。四要调整财政支出结构。加大社会保障和公共服务投资，做到应保尽保，各项保障保险金及时足额发放。 五要深化城市公益事业管理机制改革。提高服务质量，降低市民生活成本。

　　（五）转变强化政府职能，加强自身建设

　　要进一步推进依法行政。减少审批事项，放权于民，放权于市场，放权于中介组织，放权于基层，严格依法行政。

　　要进一步深化行政管理体制改革。强化经济调节、市场监管、社会管理、公共服务职能，探索新形势下社会管理新机制、新形式、新措施，提高社会管理、公共服务水平，创造宽松优良的经济发展和社会生活环境。

　　要进一步推行公开办事、阳光政务。坚持权力公开运行，决策按法定程序运作，不断扩大民主，扩大人民群众知情权、参与权、监督权，人民当家作主。

　　要进一步提高政府领导干部队伍素质和行政能力。适应城市创新转型、构建和谐社会和鹤岗经济社会大规模、高速度、科学发展的新形势新任务的要求，不断提高政府工作人员的政治素质、知识水平、行政能力、工作效率。要切实搞好廉政建设，执政为民。要培养建设真抓实干、劳而苦干、奋发有为、开拓创新的过硬作风，为鹤岗人民谋利益，为鹤岗历史负责任，干事创业，竞争图强。

　　经过五年的努力，把我市基本建成我省东部重要煤电化能源化工基地、三江平原重要绿色优质食品工业基地，接续替代产业体系初步建成，社会事业体系配套完善，城乡基础设施体系更加完备，社会保障体系功能较强，全市经济总量大幅扩张，经济质量大幅提升，财政收入大幅增加，人民生活水平大幅提升，对外开放程度大幅提高，市域经济发展后劲和市场竞争力大幅增强，自然生态环境优良，社会高度和谐文明，将我市真正建成市强民富的边疆名城。

　　三、2007年工作安排

　　今年是落实市第十次党代会精神、完成新一届政府五年任务的起步之年，做好今年的各项工作，意义重大。

今年经济社会发展的主要预期目标是：地区生产总值增长12%，财政收入按可比口径增长10%，固定资产投资增长25%，社会消费品零售总额增长10%，利用外资、进出口总额分别增长20%，城镇居民人均可支配收入增长8%，农民人均纯收入增长6%。

　　（一）务求“三年项目建设年”决战决胜，做大做强工业经济

坚定不移地实施工业立市、项目牵动战略，务求工业经济、项目建设取得重大成效。今年规模以上工业要完成增加值40亿元，增长15%；实现利税10亿元，增长11%。

集中力量推进项目建设，决战决胜三年“项目建设年”。一是精心组织好兴汇萝北30万吨玉米深加工、粉煤灰矸石空心砖二期扩建、人和米业20万吨水稻加工等去年已投产新项目达产运营。二是优质服务，促进电厂二期60万千瓦机组、兴汇宝泉岭70万吨玉米深加工、圣元乳业日处理350吨鲜奶、5000吨木糖醇等12个去年已开工项目建成投产，加快鸟山煤矿、矸石电厂2个在建项目建设。三是强力推进现有的北台百万吨化肥、中化工程集团120万吨甲醇、华能电厂三期200万千瓦机组、鲲鹏90万吨焦炭、萝北150万吨水泥、绥滨秸秆发电六个项目的运作和建设，力争化肥、甲醇、万禾园油脂二期、人和米业稻壳发电等24个新项目开工建设。四是加快煤层气开发、中国投资总公司石墨精深加工、鹤矿集团120万吨甲醇、锰矿冶炼、浙江艾可泰公司腐植酸深加工，以及180万千瓦龙江电站、7亿立方米关门嘴子水库、鹤名铁路、名山口岸二港区、绥滨松花江大桥、黑龙江大桥等一些后续项目的前期和招商工作。加快新华煤电化、鹤西高科技、宝泉岭龙头企业三个产业聚集区基础设施建设，促进产业集群发展。

　　要加大服务支持力度，提高工业经济效益和质量。一是组织金融、财政、发改委等部门，强化服务，争取政策，加大支持力度，促进鹤矿集团、宝局、鹤北局、地煤、电厂、制酒、医药、食品、建材等企业抢抓市场机遇，扩大生产，提高效益，做大做强。二是抓好禾友农药异地改造、造纸厂招商启动、工业布厂扩产改造等7个老企业的改制改造，使其焕发新的生机。三是加大政策支持力度，引导鼓励煤炭、粮食、非金属矿产资源精深加工，加快矿产伴生物、工业废弃物综合利用，发展循环经济，延长产业链条，治理环境污染，走新型工业化道路。

(二)扎实推进新农村建设，加快发展农村经济

以增加农民收入为中心，以发展农村经济、改善农村生产生活条件、发展现代农业为重点，加快新农村建设步伐。全市农业增加值要完成12.3亿元，增长7%，其中牧业增加值2.8亿元，增长7.7%；粮食总产13.37亿斤，增长8%。

　　要扎实推进新农村建设。调动和发挥农民的主体作用，支持积极性高、能力强的村镇新农村建设。加快发展农村社会事业，推进农村最低生活保障、五保户供养、农村医保等工作，提高农民生活和保障水平。要继续加大帮扶支持力度，市、县两级财政投入不低于上年水平。

　　要优化种植养殖业结构。根据市场效益和加工企业需求，扩大水稻、玉米种植面积，分别增加4.5万亩和6.5万亩。促进养殖业乘势发展，生猪、奶牛、肉牛饲养量要增长7.1%、7.1%和24%。增加10万亩有机、绿色、无公害食品种植面积，使用标识突破30个。加快农业科技进步，建立10个农业科技园区，带动农业标准化作业160万亩。

　　要提升产业化水平。全力支持兴汇粮食加工、九三油脂、宝泉岭肉业、鹤鸣米业、泰丰米业等35户省市级龙头企业的经营发展。加快建设专业化、规模化、优质化、标准化农畜产品生产基地，满足龙头企业的原料需求。创新企业与基地的利益联结机制，发展完善订单农业，促进龙头企业增产、农民增收。

　　要加强农业基础设施建设。加快推进农村公路建设。投资4000万元加强农田水利建设。完成小鹤立河水库除险扩容，争取启动德龙、江萝、团结三大灌区建设工程，建设石头河护岸、平原水库、八公里水库等9项水毁设施和水源工程、灌区节水改造工程和续建配套、市区旱田节水灌溉、人畜饮水安全等工程。大力推进农业机械化作业，更新大中型拖拉机300台、大型农机具620台。组建2个农机合作社。

　　要大力发展县域经济。加速推进县域经济工业化、产业化、城镇化和现代化建设，实施财源工程，加快项目建设，加快基础设施建设，加快社会事业发展，增强县域经济实力。要制定落实促进县域经济发展的优惠政策。

　　(三)加强基础设施建设，改善城乡生产生活条件

　　多方筹资，扩大投入，抓好以交通、水利、电力为重点的基础设施建设，以住宅和市政基础设施建设为重点，统筹城乡建设事业，加快城市化进程，扩展城市空间，增强城市功能。

　　要强化城乡基础设施建设。建设哈肇公路肇兴至名山段省级公路、通乡通村公路，完善主干公路网和农村公路网。继续强化水利基础设施，完善城市供水网建设。加强污水综合处理。加快城市电网改造，提高用电保障能力。要进一步完善市内交通、供热、供水等城镇基础设施，搞好城市配套设施建设。

　　要进一步提高城市建设管理水平。搞好住宅建设。针对当前房屋供求趋紧、房价过高的情况，要重点扩大住宅建设规模，增加供给，调控价格，改善结构，增加小户型和经济适用住房。今年完成开竣工住宅50万平方米以上，建设8栋高层住宅。基本完成沉陷区治理改造二期工程。启动城市棚户区改造工程。创新城市管理方式。严格规划控制，杜绝私批滥建。坚持完善创新城市管理长效机制，搞好环卫、河道、街巷路日常管理，实施城市交通、市场、市容、市政设施、社会治安综合治理，优化城市秩序。进一步加强社区建设，增强服务功能。扩大行业专项服务热线覆盖范围，提高服务质量，确保市民日常生活中出现的困难和诉求及时得到解决。同时，要搞好县城和重点城镇的规划、建设和管理。

　　今年要投入2.4亿元完成10件城市建设大事、实事。一是投入1500万元搞好道路建设，建设宇南路等10条主次干道，硬化62条街巷道路，维修全部街巷道路；二是投入290万元新建100座公厕；三是投入100万元改造五指山公园，规划建设麓林山城市自然公园；四是投入300万元扩大绿化规模，完成专业植树3万株、义务植树30万株；五是投入300万元加强重点河段治理，提高防洪标准；六是投入270万元实施街巷路、居民区亮化工程；七是投入2亿元完成沉陷区二期工程；八是投入800万元改造城区供水管网，新增供水用户4000户，改造50栋居民楼老化陈旧供热设施；九是投入200万元完善城市建成小区内部配套设施建设；十是投入540万元建设宇南桥。

　　筹资6.4亿元完成11件城乡基础设施建设大事、实事。一是投入1.1亿元建设环山输变电、鹤南和东郊变等区域电网；二是投入1.37亿元建设330公里农村公路；三是筹资1.83亿元扩建小鹤立河水库；四是投入9000万元建设供热三期工程，新增供热能力200万平方米；五是投入1000万元进行工业园区基础设施建设；六是投入500万元建设城市排污工程；七是投入2775万元建设17.5公里肇兴至名山段省级公路，拓宽改造4.5公里工业园道路；八是投入1200万元修建40公里村内街巷路；九是投资1260万元进行国土治理，完成治理面积1150公顷，新增耕地66公顷；十是投资1100万元，进行矿山环境治理，完成治理面积10公顷；十一是投资5500万元，进行小鹤立河、细鳞河、五号水库上游污染治理。

　　（四）扩大对外开放，提高招商引资成效和质量

　　进出口总额要实现2040万美元，实际利用外资完成240万美元；招商引资到位资金58亿元，增长20%。

　　要以扩大对俄经贸合作为重点，努力拓展对外开放领域。搞好俄罗斯“中国年”活动，巩固扩大对外经贸科技合作。以开发资源为重点，运作一批赴俄资源型、能源型大项目，突出抓好赴俄房地产开发、养殖、瓷砖生产和石油天然气开采、金矿、锰矿开发等6个项目，推动对俄经贸科技合作战略升级。扩大对外开放合作领域，重点开拓俄、日、韩等地区，支持华祥粮油、萝北天地公司等十二户企业扩大对俄、日、韩等国的粮食、木制品出口，培育一批有实力的主体企业。

　　要升级对内合作。支持域内各大企业加快发展、做强做大，发挥鹤矿集团资源实力优势，做强做大煤炭加工业，发展循环经济；发挥农垦资源实力优势，发展优质食品加工业，扩大场、县、区合作，促进农业现代化、规模化、产业化；发挥华能集团行业实力优势，做强做大煤电产业；发挥鹤北局行业资源优势，做强林产品加工业；密切同小兴安岭、三江平原市县和东部煤城的合作，资源共享，发展煤、粮、畜、木主导产业，更大程度地融入东北亚地区分工合作，扩大资源市场开发规模。

　　要以提高质量和效益为目标，全面扩大招商引资。继续坚持已有的招商政策措施，强化工作落实。继续突出领导带头招商，各县区、市直各单位主要领导要亲自带头抓招商、抓项目。扩大“走出去、请进来”规模，活跃对外联系。发挥政府与各友好地区合作联系的优势，巩固老关系，并从我市发展需要出发，在发达地区、对外开放程度高的地区选择一批新市、区建立友好关系，高层出访，政府搭台，企业唱戏，扩大招商引资成果。要更多地向上级部门汇报、争取项目资金，加快我市经济和事业发展。近几年，中央和省财政增长较快，对各项事业和基层投入支持力度加大，我市近年工资补贴、社会保障、弱势群体救助等钢性支出增加，财力紧张，各部门要加大力度向上争取支持。今年把向上争取资金项目工作进行专项考评。

　　（五）加大支持服务力度，加快发展民营经济和第三产业

　　要加快民营经济发展。民营经济是我市经济工作的战略重点，进一步发动全民创业，扩大民营经济总量；进一步动员机关干部、下岗职工、待业人员和广大人民群众，利用我市经济发展的良好机遇、宽松环境、优惠政策，积极在市场上创业发展、创业致富；进一步引导现有民营企业依据我市的发展规划，调整结构，投资资源精深加工、循环经济、高新技术产业，发展企业集团，建立现代企业制度，培植民营企业航空母舰，引领全市民营经济上规模、上档次、上水平；进一步强化对民营经济的政策资金支持、服务，优化民营经济发展环境。

　　要大力发展现代服务业。实施《商业网点规划》，建立调控网点建设工作机制，使各类市场布局合理、发展有序。培育、引进国内外知名商业集团和品牌，积极发展连锁经营，推动零售业提档升级，发展现代物流配送。发展规范信息、法律、咨询、会计、公证、经纪、代理等现代服务业。发展社区服务业，提供便民利民服务；引导文化娱乐、教育培训、体育健身、卫生保健等产业发展。深入开展“万村千乡”工程建设，改造公路两侧行政村屯100户农资、农家店。

　　要加快壮大旅游产业。加快道路、服务设施建设，打造培育“小兴安岭、黑龙江界江山水边境游”黄金线，把伊春——龙江三峡——鹤岗的旅游推向全国；搞好现有景区的经营服务，扩大知名度和辐射范围。启动创建中国优秀旅游城市活动，争取两年内迈进优秀旅游城市行列。

　　（六）做好金融财税工作，增强经济社会发展支撑能力

　　要加大财政金融对经济的支持，努力培植财源和金融市场。全面推进“诚信鹤岗”建设，营造优良的金融生态环境。财税金融战线要抢抓我市快速发展的机遇，增强财政金融筹资能力，增强对经济发展项目、社会事业项目和民生保障项目的投资支持力度，既促进全市经济社会又好又快发展，又为财税部门培植财源，为金融企业开拓市场，为人民群众增加就业岗位和提高消费水平。

　　要加大财税征收力度，确保财税收入稳定增长。连续几年的财政收入高位运行，财政增收空间缩小，使财税工作形势严峻。财税部门要加大征收执法力度，严格坚持依法征税、应收尽收。要抓好重点税源、零散税源的征管，减少跑、冒、滴、漏；要抢前抓早，坚持抓月进度、日进度，确保财政收入稳定增长。

　　要调整财政支出结构。重点向经济发展、公共服务、富民助困上倾斜，既保证经济长远加快发展，更注重保证干部群众收入增加、富民惠民项目和保障助困资金需求。重点保证在政策范围内干部工资补贴上限足额发放；保证不断提高保障水平，做到各种保障保险资金上限足额发放；保证各类各项扶贫救助资金逐年增长，保证富民惠民项目不断增加。

　　要厉行节约，控制一般性财政支出。连续几年的财政增收，增强了财政实力，提高了对经济发展、事业进步、惠民利民项目和行政经费的支持保障水平。去年以来，国家和我市调增机关事业单位人员工资，增加公务员补贴；提高离退休人员的工资和低保标准，发放低保户供热补助；增加机关事业单位经费，实行财政供养人员热费补助暗补变明补改革，扩大补助范围，提高补助标准；财政承担关停企业退休职工的医保金、增加对社会特殊弱势困难群众的救助支出、新农村建设资金等钢性支出大幅度增加。集中解决历史债务和企业遗留问题，财政压力巨大。必须坚持量入为出、“一保吃饭二保重点三是其他”的顺序，安排财政支出。坚持勤俭节约，控制一般性支出，降低行政成本和对增加经费的过高期望值，确保财政支出结构优化，应保必保。各级领导都要把更多的精力转移到创业招商、发展经济、培植财源、增加收入上来。

　　(七)协调发展社会事业，为人民富裕幸福增加更多实惠

　　要克服财力紧张的困难，积极向上争取，扩大市场招商，继续加大投入，加快社会事业发展，继续为人民群众多办好事、实事。

　　加快发展教育事业。调整优化学校布局，撤并4所中小学校。改善办学条件，加快危房改造维修。深化素质教育，提高教育标准和质量，小学、初中入学率达到100%，高中毛入学率达到60％。大力发展职业教育，培养适用型人才。提升高等教育发展水平，加快基础设施建设，全力做好师专迎评工作。

　　着力提高人民群众的健康素质。加强城乡公共卫生设施建设，完成乡镇卫生院改造任务，社区卫生服务机构人口覆盖率要达到75%。扩大农村合作医疗试点，进一步解决群众看病难问题。强化疾病预防控制，抓好重点传染病综合防治工作，健全突发公共卫生事件应急处理长效机制。完善城乡体育设施，大力发展群众体育。

　　丰富人民群众精神文化生活。大力发掘弘扬升华我市特有的抗联文化、垦荒文化、知青文化、矿区文化、边疆文化，扩大我市的文化影响力。开展丰富多彩、喜闻乐见的群众文化活动，深化文化事业改革，加强文化市场建设，依法规范文化市场秩序。丰富广播电视节目，加快广播电视数字化和农村通讯设施建设，推动农村有线电视快速发展。

　　切实加强就业再就业。增加小额担保贷款，惠及更多的下岗职工和待业人员创业就业。强化职业技能培训，搞好劳务输出；加强劳动力市场建设，规范用工就业；努力做好城镇新增劳动力、大中专毕业生、复员退伍军人、农村富余劳动力和失地农民的就业工作。全年实现新增就业2.2万人。

　　强化完善社会保障体系。完善最低生活保障体系，实现城镇低保应保尽保、按时足额发放、动态管理，实施好农村低保制度。完善社会保险体系，完善城镇职工基本养老、基本医疗和失业、工伤、生育等保险制度，扩大高风险行业工伤保险覆盖面，积极发展农村社会保险事业，扩大覆盖范围。完善社会救助体系，重点做好城镇生活无着落和流浪乞讨人员的救助工作，认真落实农村“五保”供养政策，把农村特困群众全部纳入定期救助范围。支持社会慈善、社会捐赠、社会福利、群众互助等救助活动，做好救灾救济工作。

 全力确保安全稳定。加强领导，硬化措施，综合治理，确保安全生产形势稳定。以煤矿、道路交通和消防为重点，大力建设安全设施，改善安全生产条件；健全安全生产监管体系，建立政府统一领导、部门联合执法机制，完善和落实安全生产责任制，强化企业主体责任，强化政府监管职责，严格管理，严格执法，严格责任追究；持续有效地开展专项整治行动，及时排查各类事故隐患，坚决遏制重特大事故，有效控制一般事故。加强自然灾害预测预报和防灾减灾工作。严格对药品、食品、餐饮卫生的市场监管，保障人民健康安全。加强社会治安防控体系建设，保持严打高压态势，为人民群众创造良好的治安环境。加强信访接待工作，搞好群众信访接待场所和信访信息化系统建设。

　　今年投入1亿元，完成社会事业15件大事、实事。一是投入200万元完成原鹤矿集团归属中小学2000平方米危房改造；二是投入430万元建设职教中心实习培训基地；三是筹资扩建师专图书馆和学生宿舍；四是投入350万元改扩建5所乡镇卫生院，全部完成乡镇卫生院改造任务；五是投入150万元完善社区医疗卫生服务体系建设，标准化率达50%；六是引资扩建9600平方米原兴山矿医院医疗楼；七是投入400万元全部解决社区办公服务用房；八是投入1000万元建设城市抗旱防汛指挥中心；九是投入500万元建设10个基层派出所；十是投入380万元更新改造无线电视发射台，实现中央第7套无线电视和中央1套调频广播、省交通广播落地播出；十一是投入1044万元建设市一中1万平方米教学楼；十二是投入440万元建设市一中4000平方米图书馆；十三是启动棚户区改造工程；十四是投入500万元增加公安、消防特种设备；十五是投入90万元建设三个农村客运站。

　　投入2.5亿元，完成直接惠民的15件大事、实事。一是投入200万元建立大病救助机制，使部分因病致贫的低保家庭得到医疗救助；二是投入500万元建设3500平方米鹤岗市儿童福利院；三是投入500万元建设一处老年活动中心；四是投入300万元将救助站、流浪儿童保护中心、光荣院、优抚对象疗养院、社会福利院等民政资源整合，建立鹤岗市社会福利家园；五是投入100万元将社区干部纳入社会养老保险体系；六是投入1600万元向特困低保户发放热费补贴；七是投入1100万元为200户企业1.2万企业退休职工发放基本医疗保险费；八是全面免除农村中小学学生杂费；九是投入300万元建设5000平方米廉租房；十是投入1700万元增发财政供养人员热费补助；十一是支出1.76亿元兑现机关事业单位工作人员调资和公务员津贴补助；十二是投入563万元补充政府专项救助资金，对特殊困难、弱势群体进行救助；十三是投入150万元新增30套体育路径器材及农民健身和城市社区体育健身公共设施；十四是投入142万元启动萝北县农村合作医疗；十五是投入700万元解决18个村屯1万人的饮水安全问题。

　　抓好民族、宗教、外事、监察、环保、侨务、民政、双拥、人口、国防、人防、档案、司法、人事、物价、统计、地震、残联、老龄、妇女儿童等各项事业发展。

　　（八）加强政府自身建设，提高行政能力和服务水平

　　培育和弘扬与时俱进、竞争图强的思想观念、精神状态。市十次党代会已经确定了今后五年全市经济社会发展的宏伟目标、战略措施，政府系统要大力组织落实，实现今年经济社会发展任务目标特别是搞好项目建设，完成总投入达12多亿元的51件大事、实事，将使我市经济社会发展、人民生活水平登上一个更高的台阶，意义重大，任务艰巨。全体政府工作人员要认清形势，抓住鹤岗大规模、高速度发展的机遇，适应周边地区竞相发展更加激烈市场竞争，牢固树立与时俱进的先进理念和干事创业、竞争图强的精神状态，扑下身子、豁出一头、肯于吃苦、负重前进、敢于赶超、后来居上。要求真务实，认认真真察民情，诚诚恳恳听民意，实实在在帮民富，兢兢业业保民安，为鹤岗人民的富裕幸福建功立业，为鹤岗历史的长久竞争力改革创新。

　　加强学习和实践探索，切实提高驾驭市场经济、解决实际问题的能力。政府工作任务繁重、艰难、具体，必须真抓实干、富有成效，要把自身建设的重点放在切实增强驾驭市场经济、解决实际问题的能力上。要认真学习马列主义、毛泽东思想、邓小平理论、“三个代表”重要思想，学习科学发展观的丰富内容、深刻内涵，学习市场经济理论知识，学习鹤岗经济发展中主导产业、重点项目的相关专业知识、技术，学习先进地区改革发展进步的丰富经验，学习鹤岗人民创造辉煌历史文化、卓越现实成就的精神和智慧。努力提高驾驭市场经济的能力，构建管理大项目、大产业、大企业、大集团的能力，解决社会矛盾、处理历史遗留问题、满足人民诉求、为人民办实事的能力，对外经济联系、市场招商、向上争取支持的能力，应对复杂局面、处理突发事件的能力，做群众思想政治工作、处置群体性事件的能力，自我修养、廉洁自律的能力。

　　创新管理服务机制，强化社会管理和公共服务职能。在全面履行政府“经济调节、市场监管、社会管理、公共服务”职能的同时，更加注重强化社会管理和公共服务职能。继续深化行政审批制度改革，简政放权，提高效率；进一步办好人民办事中心，创建方便群众、公开办事、规范运行的宽松环境；要强化区级政府在城市建设管理服务上的职能，强化基层基础管理；要进一步加强社区建设，完善机构，增加职能，创新机制，夯实基础，使其真正成为党和政府向人民群众提供全方位、多职能、高质量服务的最有力的基层组织；要进一步强化公安司法、城建城管、医务、房产、物业等多行业、系统的专项服务机制，及时解决市民日常生活中的困难和需求，提高服务效率和质量。要创新管理机制，完善管理制度。按照依法治市、立足治本、长治久安、高效规范的基本要求，创新城市管理公共服务领域的机制制度，对环境卫生管理、街巷道路整修管理、河道整修管理、公共设施管理等，都要建立健全专项管理条例、制度。加强项目服务中心功能建设，适应全市招商引资、全民创业、项目建设的巨大需求，对项目建设涉及的信息、立项、可研论证、报批等前期工作，提供专业服务，对项目建设中需要解决的问题和项目建成后的后续服务问题进行综合协调、全程服务，强化政府对项目建设、经济发展的服务功能，促进项目建设和企业发展。

　　自觉接受监督，加强廉政建设。政府要进一步公开办事，扩大民主，坚持阳光政务，建设廉洁政府。一要接受法律监督和民主监督。自觉接受人大的法律监督和人民群众的民主监督，定期向人大报告工作；自觉接受政协的民主监督，定期向政协通报经济和社会发展情况。要认真办理人大代表、政协委员的建议、议案、提案，虚心听取并重视民主党派、工商联和各人民团体提出的批评和意见，使人民的意愿在政府工作中真正得到体现。二要强化约束机制。开展绩效评估，建立问责制度，把行政问责与行政监察、审计监督结合起来，有责必问，有错必究。三要廉洁从政。严格遵守党的纪律，树立正确的人生观、价值观、政绩观、权利观，严格要求，防微杜渐，严守廉政防线，做清正廉洁的表率。要加强制度建设，构建教育、监督、管理并举的防治腐败机制。

　　各位代表，历史已经展开了崭新的一页，我们已经开启了鹤岗富民强市、构建和谐社会新的伟大征程，让我们在省委、省政府和市委的正确领导下，万众一心，奋发有为，干事创业，竞争图强，为鹤岗城市创新转型、人民富裕幸福、建设边疆名城做出新的、更大的贡献！
